# Nitrite is produced by elicited but not by circulating neutrophils

**DOI:** 10.1155/S0962935193000481

**Published:** 1993

**Authors:** A. G. Stewart, G. J. Dusting, R. G. Giarracca, T. Harris, Y. Lim, C. G. Sobey

**Affiliations:** 1 Microsurgery Research Centre St Vincent's Hospital Fitzroy Victoria 3065 Australia; 2 Department of Physiology University of Melbourne Victoria Australia; 3 Department of Pharmacology University of Melbourne Victoria Australia

## Abstract

The generation of nitrite (NO_2_^-^) was used as an index of the production of nitric oxide by human and rat polymorphonuclear leukocytes (PMN) and rat peritoneal macrophages. Human peripheral blood PMN did not produce significant levels of NO_2_^-^. Attempts to induce NO_2_^-^ generation in human PMN by incubation with GM–CSF (1 nM), TNFα (0.3 nM), endotoxin (1 μg/ml) or formyl-Met-Leu-Phe (100 nM) for up to 16 h were not successful. Addition of human PMN primed by GM–CSF (1 nM) to rabbit aortic ring preparations precontracted with phenylephrine had no effect on tone. In contrast to these observations, PMN, isolated from the peritoneum of oyster glycogen treated rats, generated NO_2_^-^ via a pathway sensitive to inhibition by the nitric oxide synthase inhibitor, N^G^-monomethyl L-arginine. However, peripheral blood rat PMN obtained from the same animals did not produce NO_2_^-^, even during prolonged incubation for periods of up to 16 h. It is suggested that detectable NO production by PMN requires NO synthase activity to be induced either by the process of PMN migration or by exposure to certain cytokines produced locally at the site of inflammation.


**Research Paper**

Mediators of Inflammation 2, 349–356 (1993)

### Nitrite is produced by elicited but not by circulating neutrophils

A. G. Stewart,[1,CA] G. J. Dusting,[2] R. G. Giarracca,[2] T. Harris,[1] Y. Lim[2] and C. G. Sobey[3]

[1]Microsurgery Research Centre, St Vincent's Hospital, Fitzroy, Victoria 3065, Australia, [2]Department of Physiology and [3]Department of Pharmacology, University of Melbourne, Victoria, Australia

[CA] Corresponding Author

**The generation of nitrite ($NO_2^-$) was used as an index of the production of nitric oxide by human and rat polymorphonuclear leukocytes (PMN) and rat peritoneal macrophages. Human peripheral blood PMN did not produce significant levels of $NO_2^-$. Attempts to induce $NO_2^-$ generation in human PMN by incubation with GM–CSF (1 nM), TNFα (0.3 nM), endotoxin (1 μg/ml) or formyl-Met-Leu-Phe (100 nM) for up to 16 h were not successful. Addition of human PMN primed by GM–CSF (1 nM) to rabbit aortic ring preparations precontracted with phenylephrine had no effect on tone. In contrast to these observations, PMN, isolated from the peritoneum of oyster glycogen treated rats, generated $NO_2^-$ via a pathway sensitive to inhibition by the nitric oxide synthase inhibitor, $N^G$-monomethyl L-arginine. However, peripheral blood rat PMN obtained from the same animals did not produce $NO_2^-$, even during prolonged incubation for periods of up to 16 h. It is suggested that detectable NO production by PMN requires NO synthase activity to be induced either by the process of PMN migration or by exposure to certain cytokines produced locally at the site of inflammation.**

**Key words:** Arginine analogues, Endotoxin, Formyl-Met-Leu-Phe, Macrophages, Neutrophils, Nitric oxide, Nitrite

### Introduction

Nitric oxide (NO) is a relatively long-lived free radical (half-life of 3–5 s) which is identical to, or is the active component of, endothelium derived relaxing factor.[1,2] It is claimed that NO has significant roles in haemostasis, regulation of systemic blood pressure, neurotransmission and immune responses.[3,4] Many of the physiological actions of NO appear to be mediated by activation of soluble guanylate cyclase,[4] whereas the cytotoxic activities in host defence responses appear to involve toxic interactions with Fe–S proteins.[5,6]

NO biosynthesis has been reported to occur in a variety of cell types including endothelial cells,[7] vascular smooth muscle cells,[8] adrenal gland cells,[9] Kupffer cells,[10] cerebellar neurones,[11] macrophages[12] and polymorphonuclear leukocytes (PMN).[13–16] It has recently been suggested that the NO synthase in PMN may represent a third type of isozyme which is intermediate between that of the inducible form and the calcium/calmodulin dependent constitutive form.[17,18] It is not clear whether the neutrophil enzyme is inducible or constitutive, but its activity appears to be independent of calcium/calmodulin. Much of the evidence for NO generation by PMN has come from bioassay studies. Co-incubation of rat elicited PMNs with platelets was found to significantly inhibit platelet aggregation irrespective of the stimulus for aggregation.[14,19] The inhibitory activity was prevented by the preincubation of PMNs with the NO synthase inhibitor, L-NMMA or oxyhaemoglobin, which binds and inactivates NO, suggesting that the inhibitory factor was NO. These results are in contrast to those of Schattner and coworkers[20] who found that the inhibition of platelet aggregation by co-incubation of human PMN with platelets was not prevented by oxyhaemoglobin, suggesting that the inhibition of platelet aggregation was not due to neutrophil derived NO.[20] Moreover, it has recently been suggested that platelets themselves may generate NO, and this endogenous NO may modulate platelet activity.[21] PMN derived NO has been measured by chemiluminescence,[15,16] a technique which detects inorganic $NO_2^-$ as well as NO.[22] In addition, human PMN NO generation has been measured indirectly by a spectrophotometric assay based on the reaction of the Griess reagent with $NO_2^-$, one of the stable NO decay products, which provides an index of NO generation. Wright and coworkers[16] demonstrated that unstimulated human peripheral blood PMNs were capable of generating a high basal level of $NO_2^-$.[16] Furthermore, the capacity of human peripheral blood PMN to generate NO is reported to be stimulated acutely by formyl methionyl leucyl phenylalanine (fMLP).[15] However, Keller *et al.*[23] did not observe significant NO production in activated PMN. Clearly, there are unresolved

© 1993 Rapid Communications of Oxford Ltd discrepancies in the reported generation of NO by PMN, which may relate to both the methods of detection of NO and the means of activation of the neutrophils.

In the present study the authors have examined whether human peripheral blood PMN generate significant amounts of $NO_2^-$ under basal conditions. A range of potential stimuli for PMN $NO_2^-$ production, including granulocyte macrophage colony stimulating factor (GM–CSF), tumour necrosis factor α (TNF-α) and endotoxin have also been examined. In addition, in rat elicited PMN the dependence of this $NO_2^-$ generation on protein synthesis and the relationship of NO synthesis to extravasation of neutrophils were investigated. Isolated peritoneal macrophages, which produce large amounts of $NO_2^-$, were used to establish the sensitivity of the assay of $NO_2^-$ as an index of NO generation.

### Materials and Methods

All reagents used in this study were of analytical grade or higher. Endotoxin (*E. coli* lipopolysaccharide serotype O11 B4), fMLP, penicillin-G, cycloheximide, oyster glycogen (Type 2), bovine serum albumin (cell culture tested, BSA, fraction 5), dextran T-500, Tyrode's salts (cell culture grade, endotoxin tested. Composition: HEPES 5 mM, NaCl 137 mM, D-glucose 11 mM, $NaHCO_3$ 11.9 mM, KCl 2.7 mM, $MgCl_2$ 0.26 mM and $CaCl_2$ 1.8 mM), HEPES buffer (N-2-hydroxyethylpiperazine-N-2-ethane sulphonic acid), cytochrome-c, nitro-L-arginine, superoxide dismutase, zymosan and sodium bicarbonate were all purchased from Sigma Chemical Co., St Louis, MO, USA. TNF-α was a generous gift from Dr G. Adolf, Boehringer Ingleheim, Germany. GM–CSF was a generous gift from Dr M. Rallings, Schering–Plough Pty Ltd, Sydney, Australia. Other reagents were purchased from the following sources: RPMI-1640 pH 7.4 with L-glutamine and without phenol red (Commonwealth Serum Laboratories, Parkville, Australia); phosphate buffered saline salts (Oxoid, England). All media were prepared in pyrogen-free water for injection (B.P.) (Travenol Laboratories Pty. Ltd, N.S.W., Australia); $N^G$-monomethyl-L-arginine (L-NMMA) (Calbiochem–Behring Corp., La Jolla, CA, USA); nitro-iminoethyl-L-ornithine (L-NIO, IDT, Victoria, Australia); sodium citrate (Mallinckrodt); Ficoll–Hypaque (Pharmacia, Sweden); pentobarbitone (Bomac Laboratories, N.S.W., Australia) and heparin sulphate (porcine mucous) from Fisons P/L N.S.W., Australia).

*Isolation of human peripheral blood PMN:* Buffy coats were supplied by the Red Cross blood bank, South Melbourne, Australia. PMN were isolated by dextran sedimentation of red blood cells and density gradient centrifugation through lymphoprep (5 ml) according to methods described previously in detail.[24,25] The isolated cells were stained with gentian violet and counted in an haemocytometer which indicated that >98% were PMN according to morphology and that greater than 99% of the cells were viable according to trypan blue exclusion. The PMN were resuspended in either Tyrode's buffer pH 7.4, phosphate buffered saline or phenol red free RPMI 1640 (each containing 0.25% BSA) at a concentration ranging from 2–50 × $10^6$ per ml. Since no differences were observed in the level of $NO_2^-$ generated under these different incubation conditions, the results have been pooled.

*Isolation of rat elicited PMNs and macrophages:* Peritoneal macrophages were isolated from rats according to previously described methods.[26] Briefly, male Sprague–Dawley rats (150–300 g) received an injection of oyster glycogen dissolved in phosphate buffered saline (PBS, 10 ml, 2%, i.p.). Oyster glycogen prepared at this concentration did not contain detectable endotoxin (less than 100 pg/ml), as measured by the limulus amoebocyte lysate assay (E-toxate, Sigma). After 16–20 h the rats were killed by a blow to the head. PBS (50 units/ml heparin, 30–40 ml) was injected into the peritoneal cavity. The peritoneum was gently massaged for 1 min and the lavage fluid was aspirated and centrifuged (1 000 × ***g***, 4°C, 5 min). The cell pellet was resuspended in Tyrode's buffer pH 7.4 (5 ml) and underlayered with lymphoprep (5 ml). The cell suspension was centrifuged (1 000 × ***g***, 4°C, 20 min), and the mononuclear cells were obtained from the interface between the saline and the lymphoprep solution. The mononuclear cell suspension was centrifuged (100 × ***g***, 4°C, 10 min) and an aliquot was then counted in a haemocytometer. The suspension was found to be >98% mononuclear by gentian violet staining and 99% viable by trypan blue exclusion. The cells were resuspended at a concentration of 1 × $10^6$/ml in RPMI 1640 (without phenol red, containing 0.25% BSA). One-ml aliquots of the isolated peritoneal lavage cells were dispensed into 24-well tissue culture plates. After a 2 h adherence period, the non-adherent cells were removed by aspiration.

The cell pellet from the lymphoprep separation containing PMN was resuspended in Tyrode's buffer pH 7.4 (20 ml) and centrifuged (1 000 × ***g***, 4°C, 5 min). The washing procedure was repeated once and an aliquot was stained with the gentian violet. The suspension was found to be >98% PMN. The PMNs were resuspended at a concentration of 2 × $10^6$/ml in Tyrode's buffer pH 7.4.

*Rat peripheral blood PMN:* Rats were anaesthetized with pentobarbitone (60–90 mg/kg) given subcutaneously and exsanguinated by cannulation of the carotid arteries. The blood was slowly drawn into a 10 ml syringe (containing 1 ml of sodium citrate, 3.8% w/v) and the PMN were isolated from red blood cells by dextran sedimentation and further isolated as described for human PMN.

*Rabbit isolated aortic rings:* Adult rabbits of either sex were anaesthetized by intravenous administration of saffan (3–5 ml, Glaxo Animal Health Ltd, Melbourne, Australia) and killed by exsanguination. Rings either with or without an intact endothelium were prepared and mounted in 5 ml organ baths in Krebs' bicarbonate solution as described previously.[27]

*Determination of PMN and macrophage $NO_2^-$ synthesis:* Aliquots of purified PMN (2–50 × $10^6$/ml) from human or rat peripheral blood, or of elicited rat PMN from peritoneal lavage fluid were pipetted into sterile tubes and pretreated with either the NO synthase inhibitor L-NMMA (100 μM)[14] or the protein synthesis inhibitor cycloheximide (12 μg/ml) for 50 min. Tyrode's buffer, endotoxin (1 μg/ml), GM–CSF (1 nM) or rhTNF-α (0.3 nM) was added and the PMN suspensions were incubated for 1–24 h at 37°C with gentle rocking through 45° at 22 times per min to prevent adherence. In some experiments, either buffer or fMLP (100 nM) was added during the final 5 min of incubation, the cell supernatants were obtained by centrifugation (1 000 × *g*, 4°C, 5 min) and stored at −20°C until analysis. Adherent macrophages were pretreated in an identical manner.

*Spectrophotometric assay of nitrite:* Nitrite levels from PMN or macrophage supernatants were measured using the reaction of the Griess reagent (1% sulphanilamide/0.1% napthylethylenediamine dihydrochloride/2.5% phosphoric acid) with $NO_2^-$ forming a chromophore that absorbs at 546 nm.[28] Briefly, 250 μl of sample was added to 250 μl of freshly prepared Griess reagent. Following a 5 min period to allow colour development, the absorbance was determined in a spectrophotometer (Hitachi U2000). The concentration of $NO_2^-$ was quantified by comparison with a standard curve constructed using known concentrations of $NO_2^-$ (10–100 μM). A 0.1% solution of NO was made by injecting NO gas (130 μl) from a gas tight syringe into a 13 ml glass vial containing distilled water which had been thoroughly degassed by continuous bubbling with helium for 30 min. The solution was then left open to the atmosphere for 30 min ensuring that all the NO had decayed to $NO_2^-$ and $NO_3^-$. This 0.1% solution of NO was divided into aliquots at concentrations of 10–100 μM and assayed at 546 nm, which indicated that the proportion of NO that had decayed to $NO_2^-$ was 30% at 100 μM, but this decreased to 14% in a 10 μM solution.

*Superoxide anion assay:* The generation of superoxide anion ($O_2^-$) was measured by the superoxide dismutase inhibitable reduction of cytochrome-c according to our previous studies.[25] Following 50 min pretreatment with L-NMMA (100 μM) or the vehicle, 0.25% BSA in Tyrode's buffer, PMNs were suspended at 1–2 × $10^6$/ml in Tyrode's buffer containing 80 μM cytochrome-c and stimulated with fMLP for 5 min or zymosan (400 μg/ml) for 60 min. The absorbance of the cell-free supernatants was determined in an Hitachi U2000 spectrophotometer. The generation of $O_2^-$ was calculated by the superoxide dismutase (30 U/ml) inhibitable change in absorbance at 550 nm using the differential molar extinction coefficient, 21 × $10^3$ $M^{-1}$ $cm^{-1}$.

*Statistical analyses:* All data are expressed as mean ± standard error of the mean (S.E.M.) of *n* observations. The comparison of the generation of $NO_2^-$ between the control and treated groups were analysed for statistical significance using Student's paired *t*-test. A *p* value less than 0.05 was considered to be statistically significant.

### Results

*Rat peritoneal macrophage $NO_2^-$ generation:* Macrophages isolated from rats previously treated with 2% oyster glycogen generated levels of $NO_2^-$ which were detectable for 2 h of incubation and continued to increase up to 24 h (Table 1). In peritoneal macrophages obtained from untreated animals (resident macrophages) the synthesis of $NO_2^-$ was not apparent during the first 16 h of incubation, but reached a level similar to that of elicited macrophages after 24 h of incubation. The $NO_2^-$ generation was inhibited by L-NMMA (100 μM) ($p < 0.05$, paired *t*-test) by between 36% and 66% in the various treatment groups (Table 1). Pretreatment of elicited macrophages with cycloheximide (12 μg/ml) reduced $NO_2^-$ generation ($p < 0.05$, paired *t*-test) by approximately 70% (Table 1). Investigation of the relative potencies of L-NMMA and L-NIO as inhibitors of the generation of $NO_2^-$ by resident macrophages revealed that L-NIO was considerably more potent and had a greater peak effect at the highest concentrations tested (Fig. 1).

*Human peripheral blood PMN:* A low $NO_2^-$ level was detected in supernatants obtained from human peripheral blood PMN that were incubated at 37°C for 1 h. However, the $NO_2^-$ level did not increase significantly in supernatants from cells incubated for a further 15 h (Table 2). In addition,

**Table 1.** Generation of $NO_2^-$ by rat peritoneal macrophages

| Cells | Incubation time (h) | Treatment | Concentration of $NO_2^-$ (nmol/$10^6$ cells) Control mean ± S.E.M. (n) | L-NMMA (100 μM) (mean ± S.E.M.) (n) |
|---|---|---|---|---|
| Resident macrophages | 16 | None | 0.38 ± 0.1 (3) | 0.04 ± 0.0* (3) |
| | 24 | None | 19.4 ± 4.2 (5) | 11.4 ± 3.4* (5) |
| Elicited macrophages | 16 | None | 9.2 ± 1.5 (3) | 4.9 ± 1.6* (3) |
| | 24 | None | 34.7 ± 10.6 (4) | 22.5 ± 7.1* (4) |
| | 24 | Endotoxin (1 μg/ml) | 38.6 ± 11.9 (4) | 25.3 ± 10.0* (4) |
| | 24 | Cycloheximide (12 μg/ml) | 11.8 ± 3.9** (4) | ND |

* $p < 0.05$ Student's paired t-test, compared with the corresponding value in the absence of L-NMMA.
** $p < 0.05$ Student's paired t-test, compared with the control value obtained after 24 h incubation of elicited macrophages.
ND, not done.

pretreatment with L-NMMA (100 μM) had no significant effect on the apparent $NO_2^-$ generation (Table 2). Incubation of human PMN with endotoxin (1 μg/ml), rhTNFα (0.3 nM), rhGM–CSF or fMLP (100 nM) have been shown previously to achieve maximal priming of PMN functions including the respiratory burst.[24] Therefore, human peripheral blood PMN were incubated with the optimal concentrations of these priming agents for 1 to 16 h and stimulated with fMLP for 5 min at the end of this pre-incubation period. No L-NMMA sensitive $NO_2^-$ generation was observed in PMN treated with these stimuli (Table 2). In freshly isolated human PMN, incubation with L-NMMA (100 μM) or nitro-L-arginine (NOLA, 100 μM) had no effect on the superoxide anion generation in response to either fMLP (100 nM) or zymosan (400 μg/ml) (Table 3).

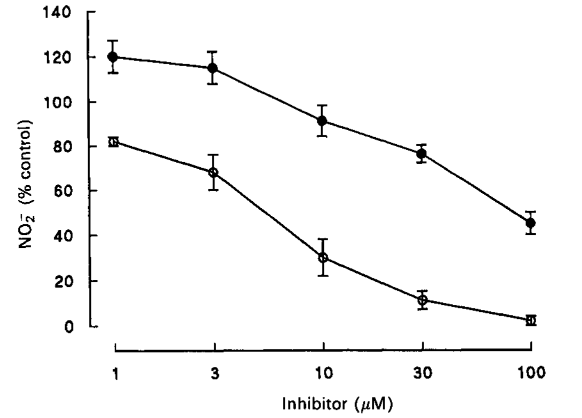

FIG. 1. Effects of pre-incubation with the L-arginine analogues, L-NMMA and L-NIO on the generation of $NO_2^-$ by rat resident peritoneal macrophages. The results are presented as the means and standard errors of the means of four observations. The control level of $NO_2^-$ detected in this 24 h incubation was 24.7 ± 6.3 nmol/$10^6$ cells. ○, L-NIO; ●, L-NMMA.

*Vasoactive effects of human peripheral blood PMN:* In rabbit aortic rings denuded of endothelium and pre-contracted with 0.1 mM phenylephrine, addition of 5 × $10^6$ PMN had no significant effect (−6.4 ± 3.0%, $n = 4$) on tone, whereas sodium nitroprusside (10 μM) reduced the phenylephrine induced tone by 101 ± 1%. The addition of PMN treated with GM–CSF (1 nM, 60 min) to the organ bath also had no effect on the tone of phenylephrine contracted preparations. By comparison, in aortic preparations containing an intact endothelium, acetylcholine (3 μM) reduced the phenylephrine induced tone by 73 ± 11%.

*$NO_2^-$ generation by rat peripheral blood and elicited peritoneal PMN:* Detectable amounts of $NO_2^-$ were observed in supernatants obtained from rat elicited PMNs after 2 h incubation and increased further up to 16 h (Table 4). Pretreatment of rat elicited PMNs with L-NMMA (100 μM) inhibited $NO_2^-$ production by approximately 60% ($p < 0.05$, paired t-test), whereas pretreatment with cycloheximide (12 μg/ml) had no inhibitory effect on $NO_2^-$ generation (Table 4). Endotoxin (1 μg/ml) caused a small increase in the amount of $NO_2^-$ generated by rat elicited PMN.

In contrast to elicited PMN, cells obtained from the peripheral blood of the same animals (i.e. those treated with oyster glycogen, i.p.) did not generate

**Table 2.** Generation of nitrite by human PMN and its inhibition by L-NMMA (100 μM)

| Treatment | Amount of $NO_2^-$ generated (nmol/$10^6$ cells) | | | | | |
|---|---|---|---|---|---|---|
| | 1 h | | 2 h | | 16 h | |
| | Control (mean ± S.E.M.) *n* | L-NMMA (mean ± S.E.M.) *n* | Control (mean ± S.E.M.) *n* | L-NMMA (mean ± S.E.M.) *n* | Control (mean ± S.E.M.) *n* | L-NMMA (mean ± S.E.M.) *n* |
| Control | 0.08 ± 0.04<br>8 | 0.15 ± 0.06<br>8 | 0.11 ± 0.07<br>8 | 0.10 ± 0.03<br>8 | 0.30 ± 0.11<br>14 | 0.23 ± 0.05<br>14 |
| Endotoxin (1 μg/ml) | 0.20 ± 0.11<br>4 | 0.14 ± 0.04<br>4 | 0.13<br>2 | 0.06<br>2 | 0.26 ± 0.09<br>8 | 0.29 ± 0.09<br>8 |
| rhTNFα (0.3 nM) | 0.07 ± 0.04<br>4 | 0.07 ± 0.03<br>3 | 0.05<br>2 | 0.07<br>2 | 0.26 ± 0.08<br>10 | 0.21 ± 0.05<br>10 |
| fMLP (100 nM) | 0.05 ± 0.03<br>3 | 0.05 ± 0.3<br>3 | 0.04<br>2 | 0.05<br>2 | 0.29 ± 0.07<br>10 | 0.25 ± 0.07<br>10 |
| rhGM-CSF (1 nM) | 0.08<br>2 | 0.08<br>2 | ND | ND | 0.19 ± 0.02<br>3 | 0.18 ± 0.04<br>3 |

**Table 3.** Effects of NO synthase inhibitors on superoxide anion generation by human PMN

| Stimulus | Amount of superoxide anion generated (nmol/$10^6$ cells) | | | |
|---|---|---|---|---|
| | Control mean ± S.E.M. (*n*) | L-NMMA 100 μM mean ± S.E.M. (*n*) | Control mean ± S.E.M. (*n*) | NOLA 100 μM mean ± S.E.M. (*n*) |
| fMLP (100 nM) | 5.50 ± 2.24 (4) | 5.68 ± 1.45 (4) | 5.17 ± 1.39 (5) | 5.07 ± 1.84 (5) |
| Zymosan (400 μg/ml) | 5.65 ± 1.54 (5) | 5.59 ± 1.32 (5) | ND | ND |

ND, not done.

**Table 4.** Generation of $NO_2^-$ by peripheral blood and peritoneal elicited PMN

| Cells | Incubation time (h) | Treatment | Concentration of $NO_2^-$ (nmol/$10^6$ cells) | |
|---|---|---|---|---|
| | | | Control (mean ± S.E.M.) (*n*) | L-NMMA (100 μM) (mean ± S.E.M.) (*n*) |
| Elicited PMN | 1 | None | 0.22 ± 0.14<br>(4) | 0.21 ± 0.16 (NS)<br>(4) |
| | 2 | None | 0.23 ± 0.09<br>(4) | 0.19 ± 0.1 (NS)<br>(4) |
| | 16 | None | 1.76 ± 0.45<br>(10) | 0.71 ± 0.24*<br>(10) |
| | 16 | Endotoxin | 2.84 ± 0.84<br>(8) | 1.64 ± 0.48*<br>(8) |
| | 16 | Cycloheximide (12 μg/ml) | 1.83 ± 0.38<br>(6) | ND |
| Peripheral blood PMN | 16 | None | 0.09 ± 0.07<br>(4) | 0.07 ± 0.07 (NS)<br>(4) |

* $p < 0.05$, paired Student's *t*-test, compared with the value obtained in the absence of L-NMMA.
NS, not significant.
ND, not done.

significant levels of $NO_2^-$. The low level of absorbance detected in the Griess reaction in these supernatants was not inhibitable by L-NMMA (100 $\mu$M). Similarly, peripheral blood PMN from untreated rats failed to generate detectable amounts of $NO_2^-$ during a 16 h incubation.

### Discussion

The present results indicate that human peripheral blood PMNs do not generate detectable levels of $NO_2^-$ during prolonged incubation. Furthermore, untreated PMN and those primed by GM–CSF did not show any NO-like bioactivity when co-incubated with pre-contracted rabbit aortic strips. The spectrophotometric assay did detect $NO_2^-$ production by rat elicited neutrophils and by macrophages, but there was no significant generation of $NO_2^-$ by rat peripheral blood PMN. In macrophages and rat elicited neutrophils, the sensitivity of the $NO_2^-$ production to inhibition by L-NMMA confirmed that the $NO_2^-$ was produced via the NO synthase pathway.

The assay used in these studies detects $NO_2^-$ concentrations as low as 0.1 nmol, a level similar to that observed in human neutrophils incubated for short periods.[29] However, there was no time-dependent increase in $NO_2^-$ production and the NO synthase inhibitor, L-NMMA had no inhibitory effect. These findings suggest that the apparent $NO_2^-$ levels detected may represent a nonspecific reaction of the Griess reagent or $NO_2^-$ production by another metabolic pathway. $NO_2^-$ generated by either rat elicited PMNs or macrophages was inhibited by incubation with L-NMMA or L-NIO (100 $\mu$M). Experiments in other cell types, including cytokine activated endothelial cells, indicate that concentrations of L-NMMA as high as 300 $\mu$M are required for near complete inhibition[30,31] and that L-NIO is considerably more potent than L-NMMA. Some of the difference in potency between these inhibitors of NO synthase may be explained by competition with L-arginine in the medium for uptake into the cell.

The failure to detect significant levels of $NO_2^-$ in human peripheral blood PMN contrasts with the findings of Wright and coworkers.[16] However, in the latter study the origin of the material reacting with the Griess reagent was not confirmed by the use of NO synthase inhibitors. Furthermore, phosphate buffered saline (PBS) was used as the incubation medium, whereas in our studies several media were used, including RPMI 1640, Tyrode's buffer and PBS; L-NMMA sensitive production of $NO_2^-$ by PMN was not observed in any of these incubation media.

Bioassay experiments have demonstrated that rat PMNs elicited into the peritoneal cavity are capable of generating NO-like biological activity as measured by an inhibition of platelet aggregation.[14] Studies by Rimele and co-workers[13] provided evidence of a neutrophil derived vasorelaxant which had properties indistinguishable from those of NO. Further studies by this group indicated that the rat elicited neutrophils generate at least ten-fold higher levels of nitrite that human peripheral blood neutrophils.[29] The present study has confirmed and extended these observations by showing that the $NO_2^-$ production in rat elicited neutrophils is reduced by NO synthase inhibitors and that more prolonged incubation of human neutrophils (up to 24 h) does not lead to accumulation of significant levels of $NO_2^-$.

*In vitro* protein synthesis does not appear to be a requirement for $NO_2^-$ production by PMN, since cycloheximide had no effect on $NO_2^-$ generation by elicited PMN. However, cycloheximide did reduce the $NO_2^-$ generation by rat macrophages, indicating that continuing protein synthesis, *in vitro*, is required for the production of NO by these cells. This differential sensitivity to cycloheximide suggests that NO synthase of PMN is induced *in vivo* and persists *in vitro*, whereas the macrophage NO synthase may turn over more rapidly and further synthesis *in vitro* is required.

The difference in the ability of rat elicited PMN and human peripheral blood PMN to generate $NO_2^-$ may represent a species difference. However, in rat peripheral blood PMN from either untreated animals or from those which received oyster glycogen 24 h earlier, there was no L-NMMA sensitive $NO_2^-$ generation, even during prolonged incubation. Thus, these findings support the suggestion that the elicitation process itself may be the stimulus for induction of the NO synthase,[29] which has been isolated from rat elicited neutrophils.[17] Alternatively, cytokine exposure during the neutrophil extravasation rather than the migration across the blood vessel wall may be the inducing stimulus. IFN-$\gamma$ and endotoxin have been shown to induce the macrophage NO synthase.[32,33] Attempts to induce $NO_2^-$ generation using endotoxin and other agents including GM–CSF, TNF$\alpha$ or fMLP were not successful. Further work is required to investigate which combination, if any, of cytokines and other inflammatory mediators is required to produce NO synthase in PMN. There is some agreement in the literature that the NO synthase inhibitors do not affect the cytostatic activity of peripheral blood PMN,[23,34] which contrasts with the discordant results regarding PMN generation of NO. It is likely that the levels of NO required for cytostasis and for detection as $NO_2^-$ are higher than those which are detected in bioassays such as platelet aggregation. Physiological roles of PMN derived NO may be restricted to regulation of function of adjacent platelets[14,35] or to locomotion of PMN themselves[36] rather than the cytotoxic role that has been established for macrophages which produce much higher amounts of NO.

It has been suggested that NO might regulate the respiratory burst, but this effect is observed only at very high concentrations.[37] The authors' studies indicate that high concentrations of NO, which are supramaximal for relaxation of isolated arteries, reduce fMLP induced superoxide anion generation by only 20% and NO does not augment the inhibitory effects of $PGE_2$.[38] Moreover, we have now shown that NO synthase inhibition in peripheral blood human PMN does not significantly affect superoxide anion production. Thus, an autocrine role for NO in the regulation of the respiratory burst in circulating PMN is unlikely.

PMN that have migrated into exudates have a higher capacity to produce superoxide anion than those in the circulation.[39,40] Both superoxide anion and NO have been ascribed important roles in host defence as cytotoxic agents. Recently, it has been suggested that the co-production of NO and superoxide anion in an acidic environment, such as that likely to be encountered at an inflammatory or ischaemic site, may result in the formation of peroxynitrite which decays to the highly reactive hydroxyl radical.[41,42] The requirement for induction of NO synthase and for priming of the respiratory burst may serve to restrict the capacity of PMN to generate such highly toxic radical species until these cells reach sites of inflammation.

In summary, we find no evidence that peripheral blood PMN generate detectable levels of $NO_2^-$, but activation of PMN during extravasation does appear to induce an NO synthase. The NO thus produced may have important roles at inflammatory sites.

### References

1. Ignarro LJ, Buga GM, Wood KS, Burns RE, Chaudhuri G. EDRF produced and released from artery and vein is NO. *Proc Natl Acad Sci (USA)* 1987; **84**: 9265–9269.
2. Palmer RMJ, Rees DD, Ashton DS, Moncada S. Nitric oxide release accounts for the biological activity of endothelium-derived relaxing factor. *Nature* 1987; **327**: 524–526.
3. Rees DD, Palmer RMJ, Schulz R, Hodson HF, Moncada S. Characterization of three inhibitors of endothelial nitric oxide synthesis *in vitro* and *in vivo*. *Br J Pharmacol* 1990; **101**: 746–752.
4. Moncada S, Palmer RMJ, Higgs EA. Nitric oxide: physiology, pathophysiology, and pharmacology. *Pharmacological Reviews* 1991; **43**: 109–141.
5. Reif DW, Simmons RD. Nitric oxide mediates iron release from ferritin. *Arch Biochem Biophys* 1990; **283**: 537–541.
6. Kwon NS, Stuehr DJ, Nathan CF. Inhibition of tumour cell ribonucleotide reductase by macrophage-derived nitric oxide. *J Exp Med* 1991; **174**: 761–767.
7. Palmer RMJ, Ashton DS, Moncada S. Vascular endothelial cells synthesise nitric oxide from L-arginine. *Nature* 1988; **333**: 664–666.
8. Wood KS, Buga GM, Burns RE, Ignarro LJ. Vascular smooth muscle-derived relaxing factor (MDRF) and its close similarity to nitric oxide. *Biochem Biophys Res Commun* 1990; **170**: 80–88.
9. Palacios M, Knowles RG, Palmer RMJ, Moncada S. Nitric oxide from L-arginine stimulates the soluble guanylate cyclase in adrenal glands. *Biochem Biophys Res Commun* 1989; **165**: 802–809.
10. Billiar TR, Curran RD, Ferrari FK, Williams DL, Simmons RL. Kupffer cell: hepatocyte cocultures release nitric oxide in response to bacterial endotoxin. *J Surg Res* 1990; **48**: 349–353.
11. Knowles RG, Palacios M, Palmer RMJ, Moncada S. Formation of NO from L-arginine in the central nervous system: a transducer mechanism for the stimulation of the soluble guanylate cyclase. *Proc Natl Acad Sci (USA)* 1989; **86**: 5159–5162.
12. Marletta MA, Youn ES, Iyengar R, Leaf CD, Wishnok JS. Macrophage oxidation of L-arginine to nitrite and nitrate: nitric oxide is an intermediate. *Biochemistry* 1988; **27**: 8706–8711.
13. Rimele TJ, Sturm RJ, Adams CM, Henry DE, Heaslip RJ, Weichman A, Grimes D. Interaction of neutrophils with vascular smooth muscle: identification of a neutrophil-derived relaxing factor. *J Pharmacol Exp Therap* 1988; **245**: 80–85.
14. McCall TB, Boughton-Smith N, Palmer RMJ, Whittle BJR, Moncada S. Synthesis of nitric oxide from L-arginine by neutrophils. *Biochem J* 1989; **261**: 293–296.
15. Schmidt HHW, Seifert R, Bohme E. Formation and release of nitric oxide from human neutrophils and HL-60 cells induced by a chemotactic peptide, platelet activating factor and leukotriene $B_4$. *FEBS Lett* 1989; **244**: 357–360.
16. Wright CD, Mulsch A, Busse R, Osswald H. Generation of nitric oxide by human neutrophils. *Biochem Biophys Res Commun* 1989; **160**: 813–819.
17. Yui Y, Hattari R, Kosuga K, Eizawa H, Hiki K, Ohkawa S, Ohnishi K. Calmodulin independent nitric oxide synthase from rat polymorphonuclear neutrophils. *J Biol Chem* 1990; **266**: 3369–3371.
18. Hiki K, Yui Y, Hattori R, Eizawa H, Kosuga K, Kawai C. Three regulation mechanisms of nitric oxide synthase. *Eur J Pharmacol* 1991; **206**: 163–164.
19. Salvemini D, De Nucci G, Gryglewski RJ, Vane JR. Human neutrophils and mononuclear cells inhibit platelet aggregation by releasing a nitric oxide-like factor. *Proc Natl Acad Sci (USA)* 1989; **86**: 6328–6332.
20. Schattner MA, Geffner JR, Isturiz MA, Lazzari MA. Inhibition of human platelet activation by polymorphonuclear leukocytes. *Br J Pharmacol* 1990; **101**: 253–256.
21. Radomski MW, Palmer RMJ, Moncada S. Characterization of the L-arginine: nitric oxide pathway in human platelets. *Br J Pharmacol* 1990; **101**: 325–328.
22. Ignarro LJ. Biological actions and properties of EDRF formed and released from artery and vein. *Circulation Research* 1989; **65**: 1–21.
23. Keller R, Keist R, Erb P *et al*. Expression of cellular effector functions and production of reactive nitrogen intermediates: a comparative study including T lymphocytes, T-like cells, neutrophil granulocytes, and mononuclear leukocytes. *Cell Immunol* 1990; **131**: 398–403.
24. Stewart AG, Harris T, De Nichilo M, Lopez AF. Involvement of leukotriene $B_4$ and platelet-activating factor in cytokine priming of human polymorphonuclear leucocytes. *Immunology* 1991; **172**: 206–212.
25. Stewart AG, Harris T. Adenosine inhibits platelet-activating factor, but not tumour necrosis factor $\alpha$-induced priming of human neutrophils. *Immunology* 1993; **78**: 152–158.
26. Lim WH, Stewart AG. Macrophage activation reduces mobilization of arachidonic acid by guinea-pig and rat peritoneal macrophages *in vitro*. *Agents Actions* 1990; **31**: 290–297.
27. Sobey CG, Dusting GJ, Stewart AG. Tumour necrosis factor-$\alpha$ augments the release of an endothelium-dependent vasoconstrictor from human polymorphonuclear leukocytes. *J Cardiovasc Pharmacol* 1992; **20**: 813–819.
28. Green LC, Wagner DA, Glogowski J, Skipper PL, Wishnok JS, Tannenbaum SR. Analysis of nitrate, nitrite, and [$^{15}$N]nitrate in biological fluids. *Anal Biochem* 1982; **126**: 131–138.
29. Rimele T, Queen T, Verghese M, Wiseman J, Holloway D, Sturm R. Interaction of inflammatory cells with vascular smooth muscle and endothelium. In: Moncada S, Higgs EA, eds. *Nitric Oxide From L-Arginine: A Bioregulatory System*. Amsterdam: Elsevier, 1990; 249–255.
30. McCall TB, Feelisch M, Palmer RMJ, Moncada S. Identification of N-iminoethyl-L-ornithine as an irreversible inhibitor of nitric oxide synthase in phagocytic cells. *Br J Pharmacol* 1991; **102**: 234–238.
31. Gross SS, Jaffe EA, Levi R, Kilbourn RG. Cytokine-activated endothelial cells express an isotype of nitric oxide synthase which is tetrahydrobiopterin-dependent, calmodulin-independent and inhibited by arginine analogues with a rank order of potency characteristic of activated macrophages. *Biochem Biophys Res Commun* 1991; **178**: 823–829.
32. Drapier JC, Wietzerbin J, Hibbs JB. Interferon-gamma and tumour necrosis factor induce the L-arginine-dependent cytotoxic effector mechanism in murine macrophages. *Eur J Immunol* 1988; **18**: 1587–1592.
33. Stuehr DJ, Gross S, Sakuma I, Levi R, Nathan CF. Activated murine macrophages secrete a metabolite of arginine with the bioactivity of EDRF and the chemical reactivity of NO. *J Exp Med* 1989; **169**: 1011–1020.
34. Kaplan SS, Billiar T, Curran RD, Zdiarski UE, Simmons RL, Basford RE. Inhibition of chemotaxis by $N^G$-monomethyl-L-arginine: a role for cyclic GMP. *Blood* 1989; **74**: 1885–1887.
35. Faint RW, Mackie IJ, Machin SJ. Platelet aggregation is inhibited by a nitric oxide-like factor released from human neutrophils *in vitro*. *Br J Haematol* 1991; **77**: 539–545.
36. Wyatt TA, Lincoln TM, Pryzwansky KB. Vimentin is transiently co-localized with and phosphorylated by cyclic GMP dependent protein kinase in formyl peptide-stimulated neutrophils. *J Biol Chem* 1991; **266**: 21274–21280.
37. Clancy RM, Leszcynska-Piziak J, Abramson SB. Nitric oxide, an endothelial cell relaxation factor, inhibits neutrophil superoxide anion production via a direct action on the NADPH oxidase. *J Clin Invest* 1992; **90**: 1116–1121.
38. Stewart AG. Inflammatory cell activation. In: Perry MA, Garlick D, eds. *Progress in Microcirculation Research*. Proceedings of the sixth Australian and New Zealand Symposium on the Microcirculation, 1991; 7–9.
39. Zimmerli W, Seligman B, Gallin JI. Exudation primes human and guinea pig neutrophils for subsequent responsiveness to the chemotactic peptide *N*-formylmethionylleucylphenylalanine and increase complement component C3bi receptor expression. *J Clin Invest* 1986; 77: 925–933.
40. Brihem G, Coble B, Stendahl O, Dahlgren C. Exudate polymorphonuclear leukocytes isolated from skin chambers are primed for enhanced response to subsequent stimulation with chemoattractant f-Met-Leu-Phe and C3-opsonized yeast particles. *Inflammation* 1988; **12**: 141–152.
41. Beckman JS, Beckman TW, Chen J, Marshall PA, Freeman BA. Apparent hydroxyl production by peroxynitrite: implications for endothelial injury from nitric oxide and superoxide. *Proc Natl Acad Sci (USA)* 1990; **87**: 1620–1624.
42. Hogg N, Darley-Usmar VM, Wilson MT, Moncada S. Production of hydroxyl radicals from simultaneous generation of superoxide and nitric oxide. *Biochem J* 1992; **281**: 419–424.

ACKNOWLEDGEMENTS. We thank the National Health and Medical Research Council and the National Heart Foundation of Australia for grant support.

**Received 27 May 1993; accepted in revised form 5 July 1993**
